# Disease progression in patients with usual interstitial pneumonia and probable UIP patterns on computed tomography with various underlying etiologies: a retrospective cohort study

**DOI:** 10.3389/fmed.2023.1246767

**Published:** 2023-10-12

**Authors:** Shuqiao Yang, Jing Wang, Di Sun, Yiran Wang, Changjiang Xue, Qiao Ye

**Affiliations:** ^1^Clinical Center for Interstitial Lung Diseases, Beijing Institute of Respiratory Medicine, Beijing Chao-Yang Hospital, Capital Medical University, Beijing, China; ^2^Department of Respiratory and Critical Care Medicine, Beijing Chao-Yang Hospital, Capital Medical University, Beijing, China; ^3^Department of Occupational Medicine and Toxicology, Beijing Chao-Yang Hospital, Capital Medical University, Beijing, China

**Keywords:** usual interstitial pneumonia, idiopathic pulmonary fibrosis, etiology, pulmonary function, transplant-free survival

## Abstract

**Background:**

Usual interstitial pneumonia (UIP) is a pattern of interstitial pneumonia that is caused by different etiologies. This study aimed to investigate the transplant-free survival (TFS) and the decline in forced vital capacity (FVC) of the patients with UIP and probable UIP patterns on CT caused by various underlying conditions.

**Methods:**

A retrospective cohort study was conducted, enrolling patients with interstitial lung disease exhibiting a CT pattern consistent with UIP or probable UIP. Clinical and prognostic data of patients categorized by the etiology were compared.

**Results:**

A total of 591 patients were included and classified into the following groups: idiopathic pulmonary fibrosis (IPF) (*n* = 320), connective tissue disease (CTD)-UIP (*n* = 229), asbestosis-UIP (*n* = 28), and hypersensitivity pneumonitis (HP)-UIP (*n* = 14). Advanced age, elevated levels of serum cytokeratin fraction 21-1 and percentage of neutrophils in bronchoalveolar lavage were observed in all groups. IPF patients showed a more rapid decline in FVC (133.9 mL/year) compared to CTD-UIP (24.5 mL/year, *p* = 0.001) and asbestosis-UIP (61.0 mL/year, *p* = 0.008) respectively. Sub-analysis of CTD-UIP revealed that patients with rheumatoid arthritis (RA)-UIP (88.1 mL/year) or antineutrophil cytoplasmic antibody-associated vasculitis (AAV)-UIP (72.9 mL/year) experienced a faster deterioration in FVC compared to those with primary Sjögren’s syndrome (pSS)-UIP (25.9 mL/year, *p* < 0.05). Kaplan–Meier curves showed that IPF had the poorest TFS (median 55.9 months), followed by HP-UIP (57.5 months), CTD-UIP (66.7 months), and asbestosis-UIP (TFS not reached). RA-UIP or AAV-UIP did not exhibit any prognostic advantages compared to IPF, while asbestosis-UIP and pSS-UIP showed better survival rates.

**Conclusion:**

Patients with UIP caused by different underlying conditions share certain common features, but the trajectories of disease progression and survival outcomes differ.

## Introduction

Usual interstitial pneumonia (UIP) is a commonly observed pattern of interstitial pneumonia characterized by heterogeneous fibrosis in the lung parenchyma, along with architectural distortion, fibroblast foci, and honeycombing. While UIP is diagnosed through pathology, extensive evidence supports the high specificity of typical and probable UIP patterns on high-resolution computed tomography (HRCT) for histopathologic UIP, eliminating the need for surgical lung biopsy. Idiopathic pulmonary fibrosis (IPF) represents the primary form of UIP with an unknown cause, but other interstitial lung diseases (ILDs), including connective tissue disease (CTD)-related, hypersensitivity pneumonitis (HP)-related, and asbestosis-related ILDs, can also exhibit a UIP pattern (1). UIP stands out from other ILD patterns, such as nonspecific interstitial pneumonia (NSIP), organizing pneumonia (OP), and lymphocytic interstitial pneumonia (LIP), due to its predominant fibrosis and minimal inflammation, which contribute to higher mortality rates, irrespective of whether it is idiopathic or secondary (2, 3).

Recent discussions have proposed the unification of UIP as a single diagnostic entity due to the presence of similar disease progression behaviors and underlying mechanisms. This unified approach has the potential to expedite antifibrotic trials for secondary UIP and trials of novel therapeutic agents (4). However, there is a scarcity of data on secondary UIP types, apart from CTD-UIP. Furthermore, previous analyses examining the disparities in survival rates between IPF and CTD-UIP have yielded inconsistent findings (5, 6). In this study, we conducted a comprehensive review of patients with UIP and probable UIP patterns on HRCT over a 10 years period at our hospital. The objective was to determine the transplant-free survival (TFS) and the decline in forced vital capacity (FVC) of the patients with different etiologies of UIP patterns and probable UIP on HRCT.

## Materials and methods

### Study design and patient selection

This study was an observational, retrospective cohort study conducted at Beijing Chao-Yang Hospital, China, which serves as a regional medical center specializing in ILD. We screened all the in-patients consecutively admitted to our hospital between January 2012 and December 2021 with newly diagnosed ILDs. Considering the limitations imposed by the local medical insurance policy and taking into account the patient’s individual preferences, hospitalized patients provide a more comprehensive set of clinical data for evaluating diseases, especially in terms of systemic organ involvement. The inclusion criterion was the presence of a UIP or probable UIP pattern on HRCT. Exclusion criteria included: (1) prior treatment with immunosuppressive or antifibrotic agents, (2) unclear diagnosis, (3) active infections, (4) malignancy or suspected malignancy at the time of diagnosis or within 1 year of diagnosis, (5) pneumothorax or pulmonary embolism at the time of diagnosis, (6) inadequate sample size for analysis, and (7) incomplete data or loss to follow-up. The study adhered to the principles of the Helsinki Declaration and its subsequent amendments and was approved by the Ethics Committee of Beijing Chao-Yang Hospital.

### Diagnosis and image evaluation

All patients included in the final analysis underwent reevaluation and discussion by a multidisciplinary team comprising a pulmonologist, rheumatologist, and radiologist. The diagnosis of IPF was based on the 2018 ATS/ERS/JRS/ALAT guideline (7). CTD was diagnosed if the patients fulfilled the established classification criteria for each specific type of CTD (8–13). Asbestosis was diagnosed using the International Labor Organization classification criteria (14), the patients included all had a history of exposure to chrysotile asbestos. HP was diagnosed following the 2020 ATS/JRS/ALAT guideline (15).

All patients underwent chest HRCT with a 1 s scan time, 1.25 mm slice thickness, and 5 mm interval from the lung apex to the base, encompassing both lungs within the field of view. The CT patterns and signs of the patients were assessed by a radiologist and pulmonologist who were blinded to the clinical data. In patients of disagreement between the pulmonologist and radiologist after the initial assessment, consultation was sought from a ILD specialist. The presence of all the following features, as outlined in the 2018 ATS/ERS/JRS/ALAT guidelines (7), defined the UIP and probable UIP patterns: heterogeneous subpleural/basal predominance, reticular abnormalities, honeycombing (reticular pattern for probable UIP), with or without traction bronchiectasis, and the absence of features inconsistent with the UIP pattern (mild ground-glass opacity may be present in probable UIP). Positive emphysema findings were visually defined as the presence of areas of low attenuation indicating a lack of a distinct alveolar wall threshold comprising more than 10% of the lung volume (16). Pulmonary hypertension (PHT) was defined as a pulmonary artery systolic pressure exceeding 35 mmHg on echocardiography (17).

### Data collection and follow-up

Demographics, smoking history, parameters of pulmonary function tests, and laboratory findings at the time of ILD diagnosis were extracted from the medical records. Prebronchodilator FVC, forced expiratory volume in the first second (FEV1), and single-breath diffusing capacity of the lung for carbon monoxide (DLCO SB) were measured according to the criteria set by the ATS/ERS (18, 19), and the percentages (%) of the normal predicted values for these parameters were recorded. The gender-age-physiology (GAP) score was calculated using sex, age, percent predicted FVC, and percent predicted DLCO, and patients were classified into GAP stages I, II, and III as previously described (20). The composite physiological index (CPI) was calculated using the formula (21): CPI = 91 − (0.65 × percent predicted DLCO) − (0.53 × percent predicted FVC) + (0.34 × percent predicted FEV1).

Blood leukocytes, neutrophils, lymphocytes, monocytes, erythrocyte sedimentation rate (ESR), C-reactive protein (CRP), immunoglobulin G (IgG), cytokeratin fraction 21-1 (CYFRA21-1), carcinoembryonic antigen (CEA), carbohydrate antigen (CA) 19-9, and CA 125 were recorded. The recommended normal ranges for each tumor marker are as follows: CYFRA21-1 ≤2.08 ng/mL, CEA ≤5 ng/mL, CA19-9 ≤37.00 U/mL, and CA125 ≤35.00 U/mL. Bronchoscopy with bronchoalveolar lavage (BAL) was performed following the guidelines of the ATS (22), and the results of the cellular analysis of the BAL fluid (BALF) were recorded. Normal BAL cell differential counts are as follows: macrophages ≥80%, lymphocytes ≤15%, neutrophils ≤3% and eosinophils ≤1%.

Longitudinal data on FVC, medication, and vital status were obtained by reviewing outpatient follow-up records and conducting phone interviews with patients or their family members. Patients were considered to have received immunosuppressive or antifibrotic therapy only if the medication was maintained for more than 6 months. For each patient, the decline in lung function was calculated as (last FVC − FVC at baseline)/duration of follow-up (in years) (23). The primary endpoint of the study was TFS, defined as the time from ILD diagnosis to the occurrence of either lung transplantation or death, up until August 2022.

### Statistical analysis

Statistical analyses were conducted using the Statistical Package for the Social Sciences (SPSS) software, version 23 (IBM Corp., Armonk, NY, United States). Categorical baseline characteristics were presented as frequencies and percentages, while continuous characteristics were reported as mean ± standard deviation or median (interquartile range, IQR). Intergroup differences for continuous variables were assessed using one-way analysis of variance or the Mann–Whitney *U* test, while categorical data were analyzed using the chi-square test. Survival analysis was performed using Kaplan–Meier curves and log-rank test. When the log-rank test was significant, Bonferroni correction for multiple comparisons was applied and the alpha level was set at 0.0083 (0.05/6 tests). Univariate and Cox proportional hazard regression analyses were then conducted to determine the predictor variables for mortality or lung transplantation, presenting hazard ratios (HRs) and 95% confidence intervals (CIs). Statistical significance was set at a *p*-value <0.05.

## Results

### Study patients

**Figure 1:**
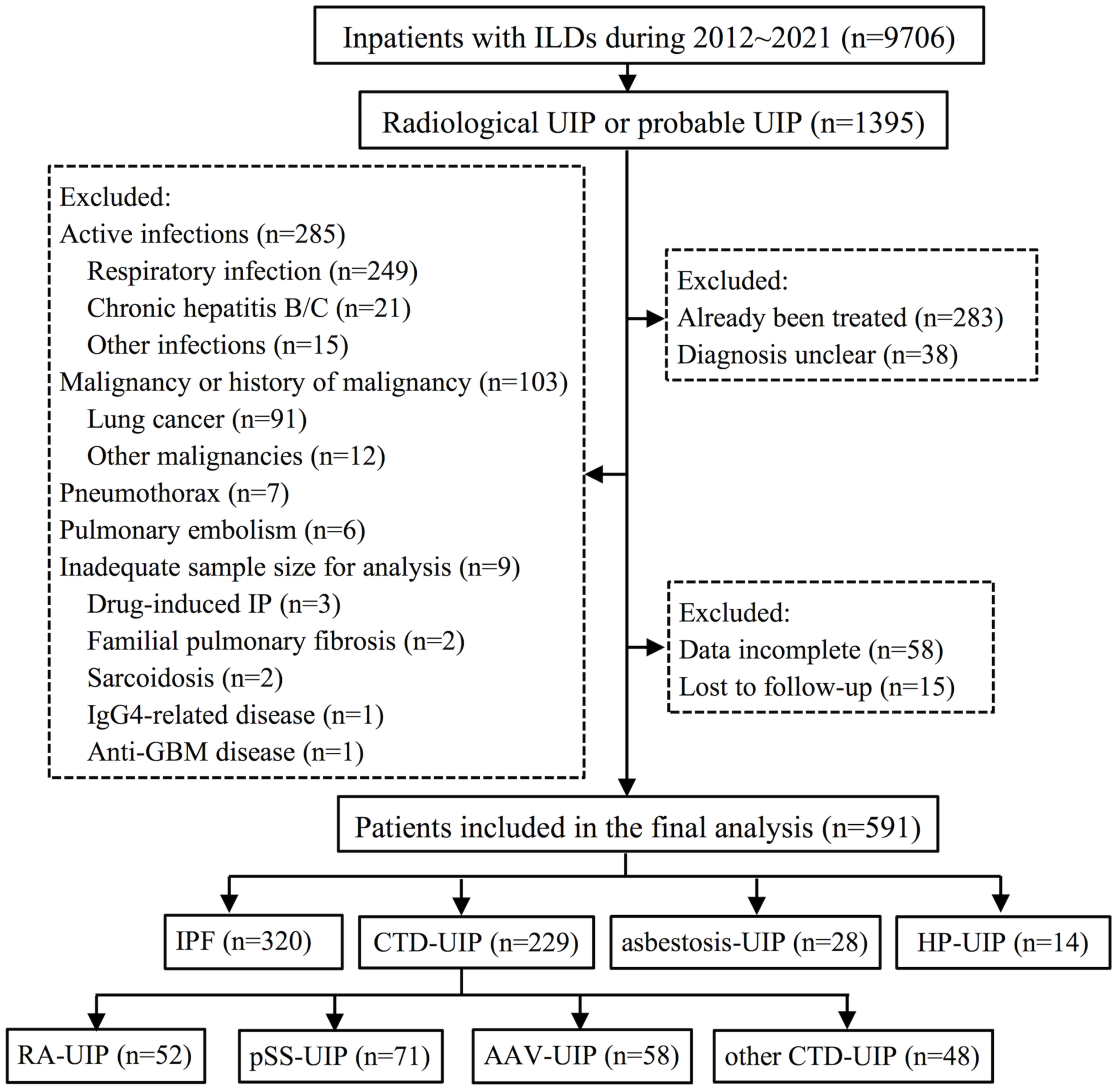
Flow diagram illustrating the patient identification process for the study. ILD, interstitial lung disease; UIP, usual interstitial pneumonia; IP, interstitial pneumonia; GBM, glomerular basement membrane; IPF, idiopathic pulmonary fibrosis; CTD, connective tissue disease; HP, hypersensitivity pneumonitis; RA, rheumatoid arthritis; pSS, primary Sjögren’s syndrome; AAV, anti-neutrophil cytoplasmic antibody-associated vasculitis.

Between 2012 and 2021, a total of 9,706 consecutive in-patients with chest HRCT detected ILD were initially screened. Among them, 1,395 patients exhibited a radiological UIP or probable UIP pattern on HRCT. Following the application of exclusion criteria, a total of 804 patients were excluded, resulting in 591 patients being included in the final analysis. The reasons for these patient’s hospitalization include dyspnea (45.5%), cough (31.6%), joint swelling or pain (7.4%), fever (4.3%), dry mouth (2.5%), abnormal chest imaging (2.0%), chest pain (1.9%), hemoptysis (1.9%), edema (1.4%), blood urine (0.8%) and fatigue (0.7%). Patients were classified into four groups: IPF (*n* = 320), CTD-UIP (*n* = 229), asbestosis associated UIP (asbestosis-UIP) (*n* = 28), and HP associated UIP (HP-UIP) (*n* = 14). Within the CTD-UIP group, there were 52 patients of rheumatoid arthritis (RA)-UIP, 71 patients of primary Sjögren’s syndrome (pSS)-UIP, 58 patients of antineutrophil cytoplasmic antibody-associated vasculitis (AAV)-UIP, and 48 patients of other CTD-UIP, which consisted of 10 patients of systemic sclerosis, 2 patients of dermatomyositis, 8 patients of mixed CTD, and 28 patients of interstitial pneumonia with autoimmune features. A detailed flow diagram illustrating the patient selection process is presented in Figure 1.

### Demographics and clinical characteristics

The average age of the patients was 65.5 years, with an average body mass index (BMI) of 24.2 kg/m^2^. Among the patients, 430 (72.8%) were male, and 351 (59.4%) had a smoking history.

A total of 436 patients (73.8%) had a UIP pattern, while 155 (26.2%) had a probable UIP pattern on HRCT. At the time of diagnosis, the average FVC was 2.8 L, with FVC% predicted, FEV1% predicted, and DLCO SB% predicted being 86.6%, 87.6%, and 53.6%, respectively. The median GAP score was 3.0 (IQR: 2.0–4.0), with 375 patients (63.5%) classified as stage I, 180 (30.4%) as stage II, and 36 (6.1%) as stage III. The mean composite physiological index (CPI) score was 40.0 (Table 1). Group comparisons showed that the CTD-UIP group had significantly fewer men (58.1% vs. 83.8%, *p* < 0.001), a lower BMI (23.5 kg/m^2^ versus 24.8 kg/m^2^, *p* < 0.001), and fewer ever-smokers (51.1% vs. 66.3%, *p* < 0.001) than the IPF group. Age, UIP/probable UIP ratio, pulmonary function, GAP score, and CPI score were comparable among the four groups at diagnosis.

**Table 1:** Baseline demographics of UIP patients in various etiology groups.

	All	IPF	CTD-UIP	Asbestosis-UIP	HP-UIP	*p*-value
N	591	320	229	28	14	
Age, years	65.5 ± 9.1	65.3 ± 8.9	65.7 ± 9.8	68.3 ± 7.3	61.9 ± 6.5	0.160
Male, *n* (%)	430 (72.8)	268 (83.8)	133 (58.1)^a^	20 (71.4)	9 (64.3)	<0.001
BMI, kg/m^2^	24.2 ± 3.7	24.8 ± 3.7	23.5 ± 3.6^a^	24.9 ± 3.8	23.8 ± 3.9	<0.001
Ever smoker, *n* (%)	351 (59.4)	212 (66.3)	117 (51.1)^a^	14 (50.0)	8 (57.1)	0.003
UIP/probable UIP	436/155	231/89	177/52	19/9	9/5	0.387
*Pulmonary function*						
FVC, L	2.8 ± 0.8	2.9 ± 0.8	2.7 ± 0.8	2.6 ± 0.9	2.8 ± 1.0	0.075
FVC% predicted	86.6 ± 20.4	85.4 ± 19.9	88.0 ± 21.3	88.1 ± 19.0	87.6 ± 20.6	0.582
FEV1% predicted	87.6 ± 19.7	87.0 ± 19.3	88.4 ± 20.3	86.8 ± 19.3	89.9 ± 21.6	0.881
DLCOsb% predicted	53.6 ± 20.1	52.6 ± 20.2	52.6 ± 19.5	60.7 ± 18.1	62.3 ± 20.7	0.083
GAP score	3.0 (2.0–4.0)	3.0 (2.0–4.0)	3.0 (2.0–4.0)	3.0 (2.0–4.0)	2.0 (1.0–2.5)	0.077
Stage I, *n* (%)	375 (63.5)	199 (62.2)	146 (63.8)	18 (64.3)	12 (85.7)	0.361
Stage II, *n* (%)	180 (30.4)	98 (30.6)	72 (31.4)	9 (32.1)	1 (7.1)	0.299
Stage III, *n* (%)	36 (6.1)	23 (7.2)	11 (4.8)	1 (3.6)	1 (7.1)	0.644
CPI score	40.0 ± 16.2	41.1 ± 16.2	39.9 ± 16.3	34.5 ± 13.0	35.5 ± 17.9	0.169

Data were presented as mean ± SD or median (IQR) or *n* (%). UIP, usual interstitial pneumonia; IPF, idiopathic pulmonary fibrosis; CTD, connective tissue disease; HP, hypersensitivity pneumonitis; BMI, body mass index; FVC, forced vital capacity; FEV1, forced expiratory volume in the first second; DLCOsb, single-breath diffusing capacity of the lung for carbon monoxide; GAP, gender-age-physiology; CPI, composite physiologic index.

a *p* < 0.0083 versus the IPF group.

Regarding laboratory findings, the neutrophils, ESR, CRP, and IgG levels in the CTD-UIP patients were significantly higher than those in the other three patients (*p* < 0.001). Among the oncomarkers, the IPF patients had higher CEA levels than the CTD-UIP group (3.3 vs. 2.4, *p* < 0.001), whereas the CTD-UIP group had higher CA 19-9 and CA125 levels than the other three patients (*p* < 0.001). Bronchoscopy with BAL was performed in 307 patients (51.9%), and the median proportions of BALF macrophages, neutrophils, lymphocytes, and eosinophils were 60.0%, 28.0%, 6%, and 1%, respectively. The median proportion of neutrophils exceeded the normal level (3%) in patients with IPF (29.0%), CTD-UIP (30.0%), asbestosis-UIP (23.5%), and HP-UIP (22.0%) (Table 2).

**Table 2:** Laboratory data of UIP patients in various etiology groups.

	All	IPF	CTD-UIP	Asbestosis-UIP	HP-UIP	*p*-value
*N*	591	320	229	28	14	
*Blood cytology*						
Leucocytes, ×10^9^/L	6.7 ± 1.9	6.6 ± 1.6	6.9 ± 2.3^b^^,^^c^	6.1 ± 1.4	5.9 ± 1.3	0.032
Neutrophils, ×10^9^/L	4.0 ± 1.6	3.8 ± 1.2	4.4 ± 2.0^a^^,^^b^^,^^c^	3.6 ± 1.0	3.1 ± 1.2	<0.001
Lymphocytes, ×10^9^/L	1.9 ± 0.7	2.1 ± 0.7	1.8 ± 0.7^a^	1.9 ± 0.6	2.1 ± 0.6	<0.001
Monocytes, ×10^9^/L	0.5 ± 0.2	0.5 ± 0.1	0.4 ± 0.2	0.5 ± 0.1	0.4 ± 0.1	0.587
ESR, mm/h	15.0 (6.0–29.0)	10.0 (3.0–18.0)	27.0 (15.0–51.0)^a^^,^^b^^,^^c^	12.0 (3.0–18.0)	6.5 (2.0–18.0)	<0.001
CRP, mg/dL	0.4 (0.2–0.7)	0.3 (0.2–0.6)	0.7 (0.3–2.4)^a^^,^^b^^,^^c^	0.4 (0.2–0.6)	0.2 (0.1–0.3)	<0.001
IgG, mg/dL	1480.2 ± 430.3	1365.2 ± 321.5	1657.5 ± 512.9^a^^,^^b^^,^^c^	1269.4 ± 204.3	1383.6 ± 222.9	<0.001
Serum oncomarkers	557 (94.2)	307 (95.9)	212 (92.6)	25 (89.3)	13 (92.9)	0.242
CYFRA21-1, ng/mL	3.1 (2.5–4.1)	3.2 (2.6–4.2)	3.0 (2.4–4.0)	2.7 (2.0–3.8)	3.1 (2.7–4.0)	0.211
CEA, ng/mL	2.9 (1.8–4.5)	3.3 (2.1–5.2)	2.4 (1.6–3.8)^a^	2.9 (1.3–3.6)	2.6 (1.1–6.5)	<0.001
CA19-9, U/mL	17.5 (7.9–43.8)	15.9 (7.0–40.2)	21.4 (11.8–68.7)^a^^,^^b^^,^^c^	14.6 (7.6–30.9)	14.5 (8.9–49.7)	0.003
CA125, U/mL	19.2 (10.4–37.3)	15.4 (10.1–31.1)	24.6 (12.4–45.3)^a^^,^^b^^,^^c^	13.4 (8.7–31.3)	10.4 (8.2–39.8)	<0.001
BALF cytology	307 (51.9)	179 (55.9)^c^	108 (47.2)^c^	6 (21.4)^a^^,^^c^	14 (100.0)	<0.001
Macrophages, %	60.0 (47.3–70.0)	60.0 (47.5–70.0)	57.0 (36.0–70.0)	72.5 (64.3–76.3)	65.0 (49.0–69.0)	0.266
Neutrophils, %	28.0 (20.0–45.0)	29.0 (21.0–45.0)	30.0 (19.0–56.0)	23.5 (15.3–28.3)	22.0 (15.0–26.0)	0.034
Lymphocytes, %	6.0 (2.0–8.4)	5.5 (2.0–8.0)^c^	5.0 (2.0–8.0)^c^	5.0 (3.8–7.0)^c^	13.0 (7.0–20.0)	0.004
Eosinophils, %	1.0 (0–3.0)	1.0 (0–3.0)	1.0 (0–3.0)	1.0 (0–3.3)	2.0 (0.5–4.0)	0.682

Data were presented as mean ± SD or median (IQR) or *n* (%). UIP, usual interstitial pneumonia; IPF, idiopathic pulmonary fibrosis; CTD, connective tissue disease; HP, hypersensitivity pneumonitis; ESR, cells erythrocyte sedimentation rate; CRP, C-reactive protein; IgG, immunoglobulin G; CYFRA21-1, cytokeratin fraction 21-1; CEA, carcinoembryonic antigen; CA, carbohydrate antigen; BALF, bronchoalveolar lavage fluid.

a *p* < 0.0083 versus the IPF group.

b *p* < 0.0083 versus the AS-UIP group.

c *p* < 0.0083 versus the HP-UIP group.

### Follow-up data

Among all patients with UIP, the frequencies of complicated emphysema or pulmonary hypertension were 31.1% and 17.9%, respectively, with no significant differences between the patients. The median follow-up duration was 46.4 months, during which immunosuppressive medications were administered to 213 patients (36.0%), antifibrotic medications to 125 patients (21.2%), and lung transplants were performed in 9 patients (1.5%). The median annual decline in FVC was 76.0 mL. The 1 year, 3 years, and 5 years TFS rates were 90.0%, 71.0%, and 52.3%, respectively. Comparing the patients, antifibrotic therapy was more frequently used in patients with IPF compared to those with CTD-UIP (29.7% vs. 12.2%, *p* < 0.001) and asbestosis-UIP (29.7% vs. 0, *p* < 0.001). The median FVC decline in the IPF group (133.9 mL/year) was more rapid than the CTD-UIP (24.5 mL/year, *p* = 0.001) and the asbestosis-UIP groups (61.0 mL/year, *p* = 0.008) (Table 3).

**Table 3:** Comorbidities, treatment, FVC decline and transplant-free survival of UIP patients in various etiology groups.

	All	IPF	CTD-UIP	Asbestosis-UIP	HP-UIP	*p*-value
*N*	591	320	229	28	14	
Emphysema, *n* (%)	184 (31.1)	112 (35.0)	63 (27.5)	7 (25.0)	2 (14.3)	0.111
Pulmonary hypertension, *n* (%)	106 (17.9)	56 (17.5)	43 (18.8)	6 (21.4)	1 (7.1)	0.679
Follow-up time, month	46.4 (23.5–68.1)	45.9 (24.2–66.9)	47.9 (21.9–71.1)	51.8 (39.7–72.6)	40.4 (17.1–53.4)	0.328
*Treatment, n (%)*						
Immunosuppressive therapy	213 (36.0)	14 (4.4)	187 (81.7)^a^^,^^b^	0 (0)	12 (85.7)^a^^,^^b^	<0.001
Antifibrotic therapy	125 (21.2)	95 (29.7)	28 (12.2)^a^	0 (0)^a^	2 (14.3)	<0.001
Lung transplantation	9 (1.5)	6 (1.9)	3 (1.3)	0 (0)	0 (0)	0.867
ΔFVC, mL/year	76.0 (−7.7–203.4)	133.9 (39.3–269.1)	24.5 (−72.8–158.9)^a^	61.0 (37.9–119.8)^a^	86.0 (−186.1–213.5)	0.003
*Cumulative TFS, %*						
1 year	90.0%	89.4%	89.5%	96.4%	92.9%	—
3 years	71.0%	68.3%	71.6%	88.7%	82.5%	—
5 years	52.3%	48.1%	55.2%	78.3%	47.2%	—

Data were presented as median (IQR) or *n* (%). UIP, usual interstitial pneumonia; IPF, idiopathic pulmonary fibrosis; CTD, connective tissue disease; HP, hypersensitivity pneumonitis; FVC, forced vital capacity; TFS, transplant-free survival.

a *p* < 0.0083 versus the IPF group.

b *p* < 0.0083 versus the AS-UIP group.

### Subgroup analysis

A subgroup analysis was conducted specifically for patients with CTD-UIP. Most demographic and laboratory characteristics were comparable among patients with RA-UIP, pSS-UIP, AAV-UIP, and other CTD-UIP. However, there were notable differences in age and levels of blood inflammatory markers, including leukocytes, neutrophils, ESR, and CRP levels, which were higher in AAV-UIP patients. The rates of decline in FVC varied among the patients. Both RA-UIP (88.1 mL/year) and AAV-UIP (72.9 mL/year) patients exhibited significantly faster FVC decline compared to pSS-UIP (25.9 mL/year) and other CTD-UIP patients (20.7 mL/year), with a significance level of *p* < 0.05 (Supplementary Table S1).

Additional comparisons were conducted between patients with or without emphysema, and those with or without PHT in the IPF and CTD-UIP groups. Patients with emphysema presented slower median annual FVC decline than those without emphysema, both in the IPF group (50.7 mL/yr vs. 177.3 mL/yr, *p* = 0.047) and the CTD-UIP group (-35.6 mL/yr vs. 38.9 mL/yr, *p* = 0.024). Patients with PHT, however, presented significantly shorter TFS than those without PHT in the IPF group (35.9 vs. 65.2 month median TFS, *p* < 0.001) and the CTD-UIP group (28.5 vs. 80.9 month median TFS, *p* < 0.001) (see Supplementary Tables S2,S3).

### Transplant-free survival and risk factors

**Figure 2:**
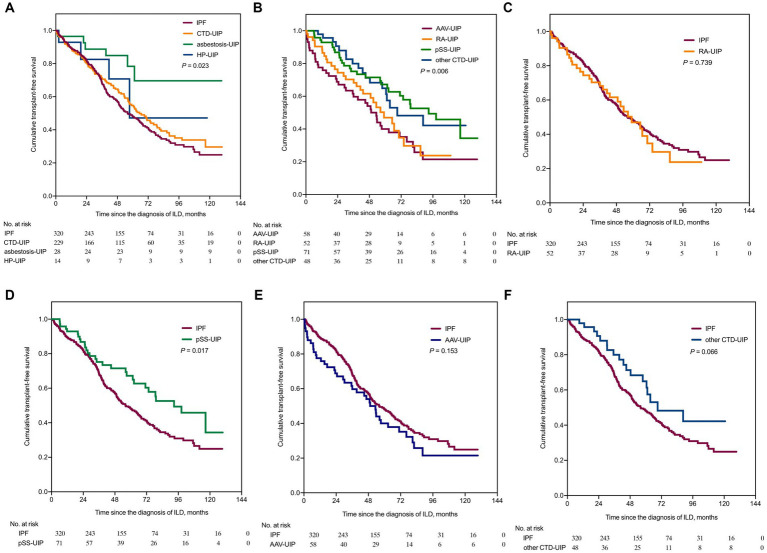
Kaplan–Meier survival curves comparing transplant-free survival among patients with UIP of various etiologies. TFS, transplant-free survival. **(A)** TFS comparison between IPF, CTD-UIP, asbestosis-UIP, and HP-UIP (*p* = 0.023). **(B)** TFS comparison among RA-UIP, pSS-UIP, AAV-UIP, and other CTD-UIP (*p* = 0.006). **(C)** TFS comparison between IPF and RA-UIP [HR = 1.069 (95% CI: 0.715, 1.597), *p* = 0.739, compared to IPF]. **(D)** TFS comparison between IPF and pSS-UIP [HR = 0.624 (95% CI: 0.446, 0.872), *p* = 0.017, compared to IPF]. **(E)** TFS comparison between IPF and AAV-UIP [HR = 1.286 (95% CI: 0.882, 1.875), *p* = 0.153, compared to IPF]. **(F)** TFS comparison between IPF and other CTD-UIP, [HR = 0.638 (95% CI: 0.425, 0.958), *p* = 0.066, compared to IPF]. IPF, idiopathic pulmonary fibrosis; CTD, connective tissue disease; UIP, usual interstitial pneumonia; HP, hypersensitivity pneumonitis; RA, rheumatoid arthritis; pSS, primary Sjögren’s syndrome; AAV, anti-neutrophil cytoplasmic antibody-associated vasculitis; HR, hazard ratio; CI, confidence interval.

The Kaplan–Meier curves in Figure 2 illustrate the differences in TFS among UIP patients with different etiologies. Statistically significant distinctions were observed among the IPF, CTD-UIP, asbestosis-UIP, and HP-UIP patients (*p* = 0.023) (Figure 2A). The IPF group exhibited the poorest prognosis (median TFS: 55.9 months), followed by the CTD-UIP group (median TFS: 66.7 months) and HP-UIP group (median TFS: 57.5 months). The asbestosis-UIP group demonstrated the best survival, with the median TFS not being reached. Pairwise comparisons revealed significant differences between the IPF and asbestosis-UIP patients (log-rank *p* < 0.001). While the difference did not reach statistical significance after Bonferroni correction, a marginal trend towards better survival was observed in the CTD-UIP group compared to the asbestosis-UIP group (*p* = 0.017).

Furthermore, differences in TFS were observed within the CTD-UIP patients (*p* = 0.006) (Figure 2B). The AAV-UIP group (median TFS: 49.3 months) and RA-UIP group (median TFS: 59.0 months) had the poorest prognoses, while the pSS-UIP group (median TFS: 92.8 months) and other CTD-UIP group (median TFS: 69.1 months) exhibited better TFS. A significant difference was found between the AAV-UIP and pSS-UIP patients (log-rank *p* < 0.001), and a trend towards better survival was observed in the pSS-UIP group compared to the RA-UIP group (*p* = 0.017). When the IPF group was used as a control, neither the RA-UIP nor AAV-UIP patients showed a survival advantage, whereas a significant survival advantage was found in the pSS-UIP group (*p* = 0.017), and a marginal survival advantage was observed in the other CTD-UIP group (*p* = 0.066) (Figures 2C–F).

Univariate analysis indicated that age (HR 1.043, *p* < 0.001) and PHT (HR 1.769, *p* = 0.002) were risk factors, while female sex, probable UIP (HR 0.433, *p* < 0.001, compared with UIP), FVC% predicted (HR 0.971, *p* < 0.001), DLCO SB% predicted (HR 0.971, *p* < 0.001), pSS-UIP (HR 0.599, *p* = 0.011, compared with IPF), and asbestosis-UIP (HR 0.320, *p* = 0.006, compared with IPF) were protective factors for mortality or lung transplant. Cox hazard modeling demonstrated that the impact of etiology on survival persisted even after adjusting for age, sex, radiological patterns, pulmonary function, and PHT at the time of diagnosis (Table 4).

**Table 4:** Risk factors associated with death or lung transplantation in UIP patients.

Covariates	Univariable analysis		Multivariable analysis	
	HR (95% CI)	*p*-value	HR (95% CI)	*p*-value
Age	1.038 (1.024–1.051)	<0.001	1.043 (1.025–1.060)	<0.001
*Sex*				
Male	1.000 (reference)	—	—	—
Female	0.649 (0.496–0.849)	0.002	0.981 (0.632–1.525)	0.933
BMI	0.981 (0.950–1.013)	0.235	—	—
Smoking history	1.244 (0.988–1.566)	0.063	1.106 (0.771–1.587)	0.583
*Radiological pattern*				
UIP	1.000 (reference)	—	—	—
Probable UIP	0.433 (0.315–0.596)	<0.001	0.664 (0.434–0.954)	0.049
FVC% predicted	0.971 (0.964–0.977)	<0.001	0.977 (0.968–0.987)	<0.001
DLCOsb% predicted	0.971 (0.965–0.978)	<0.001	0.986 (0.977–0.996)	0.004
Emphysema	0.909 (0.710–1.164)	0.449	—	—
PHT	2.319 (1.779–3.023)	<0.001	1.769 (1.236–2.533)	0.002
*Etiology*				
IPF	1.000 (reference)	—	—	—
RA-UIP	1.070 (0.723–1.584)	0.735	1.003 (0.581–1.733)	0.991
pSS-UIP	0.599 (0.404–0.888)	0.011	0.624 (0.423–0.987)	0.047
AAV-UIP	1.286 (0.910–1.818)	0.155	0.804 (0.462–1.400)	0.441
Other CTD-UIP	0.631 (0.388–1.024)	0.062	0.563 (0.307–1.033)	0.063
Asbestosis-UIP	0.320 (0.142–0.722)	0.006	0.154 (0.038–0.630)	0.008
HP-UIP	0.828 (0.340–2.014)	0.677	1.953 (0.735–4.873)	0.170

HR, hazard ratio; CI, confidence interval; BMI, body mass index; UIP, usual interstitial pneumonia; FVC, forced vital capacity; DLCOsb, single-breath diffusing capacity of the lung for carbon monoxide; PHT, pulmonary hypertension; IPF, idiopathic pulmonary fibrosis; RA, rheumatoid arthritis; pSS, primary Sjögren’s syndrome; AAV, anti-neutrophil cytoplasmic antibody associated vasculitis; CTD, connective tissue disease; HP, hypersensitivity pneumonitis.

## Discussion

This study focused on patients with ILD exhibiting UIP and probable UIP patterns on HRCT. Our findings revealed significant heterogeneity in disease progression and some shared characteristics among UIP patients with different etiologies.

In the current study, we observed significant heterogeneity in FVC decline rates and TFS among patients with UIP of different etiologies. Asbestosis, characterized by irregular and/or linear opacities with basal preponderance, ground-glass opacities, and mosaic attenuation accompanied by pleural abnormalities, is caused by repeated asbestos inhalation (24). Our recently published data showed that 9.8% (20/204) of patients with asbestosis manifested a UIP pattern on HRCT (25). Limited data are available regarding the survival of asbestosis-UIP patients. In our study, asbestosis-UIP patients demonstrated a slowly progressive disease course and better survival outcomes compared to IPF patients, despite the two groups being indistinguishable in terms of clinical and laboratory features. Another disease entity in our cohort, UIP associated with pSS also exhibited a prognostic advantage over IPF. Pathological comparisons revealed that fibroblast foci, a characteristic feature of IPF, are infrequent and sporadically observed in pSS-UIP and asbestosis patients, whereas interstitial inflammation, plasma cell infiltration, lymphoid follicles with germinal centers, cysts, bronchiolitis, and pleuritis were significantly more pronounced in the lungs of pSS-UIP patients (26, 27). Recent studies utilizing artificial intelligence to quantify fibroblast foci have demonstrated that a large area occupied by fibroblast foci predicts a poor prognosis in IPF (28). Similarly, data from patients with chronic HP have indicated that the presence of fibroblast foci is an independent predictor of time to death or transplantation (29). Thus, we consider the number of fibroblastic foci to be at least one of the crucial factors contributing to the differences in fibrosis progression and mortality among patients with UIP. However, the factors influencing the presence and number of fibroblastic foci remain unknown.

In contrast to pSS-UIP, both the decline rate in FVC and TFS in patients with RA-UIP and AAV-UIP were similar to those observed in IPF. This finding could help explain the conflicting evidence regarding prognostic differences between CTD-UIP and IPF, as the composition of CTD patients varies across studies (5, 6). Studies comparing survival between RA-UIP and IPF, as well as AAV-UIP and IPF, have reported results consistent with our findings (30, 31). Interestingly, in a study by Watanabe et al. (32), while the decline in lung volume and median survival time were similar between AAV-UIP and IPF patients, the causes of death differed. AAV-UIP patients more frequently experienced deaths related to anti-immune therapy (infectious complications) or vasculitis itself (alveolar hemorrhage and potentially cardiovascular disease), whereas in IPF, most deaths were related to respiratory system issues. Nevertheless, considering the potential benefits, early initiation of antifibrotic therapy may be considered in patients with RA and AAV exhibiting a UIP pattern. Moreover, due to the evident heterogeneity, it may be inappropriate to treat CTD-ILD as a unified entity when conducting analyses related to ILD progression and survival. Furthermore, we recommend considering the specific etiology before conducting clinical trials, as therapeutic effects may diverge among patients with different progression patterns.

It is worth to mention that the annual decline rate of FVC observed in IPF patients from the current study appears to be slower than that reported in several large-scale clinical trials. In the TOMORROW trial, the annual decline rate of FVC was 60 mL in the 150 mg twice daily nintedanib group and 190 mL in the placebo group (33). In the INPULSIS trial, adjusted rate of decline in FVC was 112.4 mL/year with nintedanib and 223.3 mL/year with placebo (34). We noticed that the baseline FVC% (85.4%) of the patients in this cohort was higher than that in the TOMORROW (80.2%) and INPULSIS trials (80%). Besides, a portion of patients in the current study had received antifibrotic treatments during the study period. These could be the possible reasons for the different FVC decline rate in our study.

Despite the heterogeneities, the clinical and laboratory data of patients with UIP from various causes display remarkable similarities, shedding light on the shared underlying mechanisms of this pattern. The findings of our current study demonstrate that the median age of patients in each group exceeded 60 years, indicating a probable correlation between the UIP pattern and aging. Advanced age has long been recognized as a risk factor for IPF. Epidemiological evidence consistently reveals a higher prevalence and incidence of IPF with increasing age (35), while mechanistic investigations have underscored the crucial role of age-related processes, such as telomere attrition, cellular senescence, stem cell exhaustion, and disrupted intercellular communication, in the development of IPF (36). Similarly, in secondary ILDs, studies have reported older age in ILDs associated with CTD compared to CTD without ILD (37, 38), as well as in chronic HP compared to acute HP (39). In a recent study by Laurent et al. (40), a significant positive correlation was found between definite or possible UIP patterns and age in individuals exposed to asbestos. However, previous studies have yielded inconsistent findings regarding age differences between patients with UIP and NSIP (30, 41). Thus, it is plausible that the association with aging might be more closely related to the fibrosis behavior itself rather than specific ILD patterns. Another noteworthy shared characteristic among patients with UIP is the presence of elevated levels of CYFRA21-1 in the serum and neutrophils in BALF. The significance of CYFRA21-1 in ILDs has been increasingly recognized in recent years. CYFRA21-1 is a cytoskeletal protein expressed in type 1 and type 2 pneumocytes, as well as respiratory bronchiolar epithelial cells, and its release is triggered by lung injury (42). Notably, Molyneaux et al. (43) discovered that baseline concentrations of CYFRA21-1 could potentially identify individuals at risk of disease progression within 12 months and predict overall mortality in patients with IPF. In our study, we observed comparably elevated levels of CYFRA21-1 in both IPF and secondary UIP patients. Similarly, previous investigations have underscored the importance of neutrophils in the immune pathogenesis of IPF (44, 45), and our data revealed that BALF neutrophils were equally elevated in IPF and secondary UIP, indicating analogous immune pathogenesis in UIP caused by various underlying conditions. However, the elevation in BALF neutrophils does not appear to be exclusive to UIP (46). One possible explanation is that BALF neutrophils are associated with lung fibrosis in general rather than specifically with the UIP pattern, which is supported by the increase in neutrophils and decrease in lymphocytes observed in the BALF during the transition from acute to chronic hypersensitivity pneumonitis (39).

The similarities between IPF and other ILDs with “IPF-like” behaviors gave birth to the concept of progressive pulmonary fibrosis (PPF), which indeed, has its clinical values, especially on drug development and application. However, precise and personalized medication should not be neglected. Thus, “smarter lumping and smarter splitting” maybe advocated for these patients (47, 48). As for UIP patients, on the one hand, exploring the common biological pathways during UIP development may provide broader directions for therapy; on the other hand, tailored therapies based on precision medicine urge deeper investigations for the causes of heterogeneity in disease progression and prognosis of these patients.

The current study had several limitations that should be mentioned. Firstly, being a retrospective single-center study, it may be prone to selection bias, and the number of asbestosis-UIP and HP-UIP patients was relatively small. In the real world, the cases of HP-UIP and asbestosis are limited, a previous published study by our team showed that 9.8% (20/204) of patients with asbestosis and 10.8% (8/74) of patients with fibrotic HP manifested a UIP pattern on HRCT (25). However, this limitation may affect the accuracy of our assessments and result in an insufficient statistical power to detect existing differences, thus, some of the results must be interpretated with caution. Secondly, not all patients underwent a multidisciplinary team (MDT) assessment at the time of ILD diagnosis, although we conducted retrospective MDT discussions for each patient and excluded those with unclear diagnoses, there remains a possibility of misjudgments regarding the etiology in a few patients. Thirdly, the majority of the pathological results were derived from transbronchial lung biopsies, which prevented the comprehensive analysis of pathological features in this study. Fourthly, the assessment of the likelihood of developing PHT relied on echocardiography rather than right heart catheterization (RHC), which may have compromised accuracy. However, it is important to note that echocardiography offers the advantage of being non-invasive. RHC is an invasive procedure associated with potential risks for patients, and its benefits for patients with ILD are limited. Studies have demonstrated that in patients with PHT, the measurement of tricuspid regurgitation velocity (TRV) correlates significantly with pulmonary artery systolic pressure (PASP) obtained through RHC. Echocardiography has consistently proven to be a reliable method for diagnosing PHT (49). Last, the cause of death was not analyzed due to the fact that most deaths occurred outside the hospital, thus the specific reasons for the observed survival disparities among patients remain unknown.

In conclusion, this respective cohort study demonstrates that patients with UIP stemming from various etiologies exhibit common features such as advanced age, elevated serum levels of CYFRA21-1, and increased neutrophil counts in BALF. However, there are notable differences in the decline rate of FVC and TFS among these patients. Specifically, UIP associated with asbestosis and pSS-UIP exhibit slower disease progression and improved survival rates compared to IPF.

## Data availability statement

The original contributions presented in the study are included in the article/Supplementary material, further inquiries can be directed to the corresponding author.

## Ethics statement

The studies involving humans were approved by The Ethics Committee of Beijing Chao-Yang Hospital. The studies were conducted in accordance with the local legislation and institutional requirements. Written informed consent for participation was not required from the participants or the participants’ legal guardians/next of kin in accordance with the national legislation and institutional requirements.

## Author contributions

QY: conceptualization and methodology. SY: data curation, formal analysis, and writing-original draft preparation. JW: writing-reviewing and editing. DS: software and validation. YW: visualization and investigation. CX: project administration and supervision. All authors contributed to the article and approved the submitted version.

## Figures and Tables

**Table:** 

AAV	Antineutrophil cytoplasmic antibodies associated vasculitis
BALF	Bronchoalveolar lavage fluid
BMI	Body mass index
CA	Carbohydrate antigen
CEA	Carcinoembryonic antigen
CIs	Confidence intervals
CPI	Composite physiological index
CRP	C-reactive protein
CTD	Connective tissue disease
CYFRA21-1	Cytokeratin fraction 21-1
DLCOsb	Single-breath diffusing capacity of the lung for carbon monoxide
ESR	Erythrocyte sedimentation rate
FEV1	Forced expiratory volume in the 1st second
FVC	Forced vital capacity
GAP	Gender-age-physiology
HP	Hypersensitivity pneumonitis
HRs	Hazard ratios
HRCT	High-resolution computed tomography
IgG	Immunoglobulin G
ILD	Interstitial lung diseases
IPF	Idiopathic pulmonary fibrosis
IQR	Interquartile range
MDT	Multidisciplinary team
NSIP	Nonspecific interstitial pneumonia
PASP	Pulmonary artery systolic pressure
PHT	Pulmonary hypertension
PPF	Progressive pulmonary fibrosis
pSS	Primary Sjögren’s syndrome
RA	Rheumatoid arthritis
RHC	Right heart catheterization
TFS	Transplant-free survival
TRV	Tricuspid regurgitation velocity
UIP	Usual interstitial pneumonia

## References

[1] Raghu G Remy-Jardin M Richeldi L Thomson CC Inoue Y Johkoh T. Idiopathic pulmonary fibrosis (an update) and progressive pulmonary fibrosis in adults: an official ATS/ERS/JRS/ALAT clinical practice guideline. Am J Respir Crit Care Med. (2022) 205:e18–47. doi: 10.1164/rccm.202202-0399ST, PMID: 35486072 PMC9851481

[2] Nagai S Kitaichi M Itoh H Nishimura K Izumi T Colby TV. Idiopathic nonspecific interstitial pneumonia/fibrosis: comparison with idiopathic pulmonary fibrosis and BOOP. Eur Respir J. (1998) 12:1010–9. doi: 10.1183/09031936.98.12051010, PMID: 9863989

[3] Kim HC Lee JS Lee EY Ha Y-J Chae EJ Han M. Risk prediction model in rheumatoid arthritis-associated interstitial lung disease. Respirology. (2020) 25:1257–64. doi: 10.1111/resp.13848, PMID: 32441061 PMC7615175

[4] Selman M Pardo A Wells AU. Usual interstitial pneumonia as a stand-alone diagnostic entity: the case for a paradigm shift? Lancet Respir Med. (2023) 11:188–96. doi: 10.1016/S2213-2600(22)00475-1, PMID: 36640788

[5] Park JH Kim DS Park I-N Jang SJ Kitaichi M Nicholson AG. Prognosis of fibrotic interstitial pneumonia: idiopathic versus collagen vascular disease-related subtypes. Am J Respir Crit Care Med. (2007) 175:705–11. doi: 10.1164/rccm.200607-912OC 17218621

[6] Alhamad EH. Clinical characteristics and survival in idiopathic pulmonary fibrosis and connective tissue disease-associated usual interstitial pneumonia. J Thorac Dis. (2015) 7:386–93. doi: 10.3978/j.issn.2072-1439.2014.12.40, PMID: 25922716 PMC4387436

[7] Raghu G Remy-Jardin M Myers JL Richeldi L Ryerson CJ Lederer DJ. Diagnosis of idiopathic pulmonary fibrosis. An official ATS/ERS/JRS/ALAT clinical practice guideline. Am J Respir Crit Care Med. (2018) 198:e44–68. doi: 10.1164/rccm.201807-1255ST, PMID: 30168753

[8] Aletaha D Neogi T Silman AJ Funovits J Felson DT Bingham CO. 2010 Rheumatoid arthritis classification criteria: an American College of Rheumatology/European League Against Rheumatism collaborative initiative. Arthritis Rheum. (2010) 62:2569–81. doi: 10.1002/art.27584, PMID: 20872595

[9] van den Hoogen F Khanna D Fransen J Johnson SR Baron M Tyndall A. 2013 classification criteria for systemic sclerosis: an American College of Rheumatology/European League Against Rheumatism collaborative initiative. Arthritis Rheum. (2013) 65:2737–47. doi: 10.1002/art.38098, PMID: 24122180 PMC3930146

[10] Bohan A Peter JB. Polymyositis and dermatomyositis (first of two parts). N Engl J Med. (1975) 292:344–7. doi: 10.1056/NEJM197502132920706 1090839

[11] Vitali C Bombardieri S Jonsson R Moutsopoulos HM Alexander EL Carsons SE. Classification criteria for Sjögren’s syndrome: a revised version of the European criteria proposed by the American-European Consensus Group. Ann Rheum Dis. (2002) 61:554–8. doi: 10.1136/ard.61.6.554, PMID: 12006334 PMC1754137

[12] Sharp GC Irvin WS Tan EM Gould RG Holman HR. Mixed connective tissue disease-an apparently distinct rheumatic disease syndrome associated with a specific antibody to an extractable nuclear antigen (ENA). Am J Med. (1972) 52:148–59. doi: 10.1016/0002-9343(72)90064-2 4621694

[13] Fischer A Antoniou KM Brown KK Cadranel J Corte TJ du Bois RM. An official European Respiratory Society/American Thoracic Society research statement: interstitial pneumonia with autoimmune features. Eur Respir J. (2015) 46:976–87. doi: 10.1183/13993003.00150-2015, PMID: 26160873

[14] American Thoracic Society. Diagnosis and initial management of nonmalignant diseases related to asbestos. Am J Respir Crit Care Med. (2004) 170:691–715. doi: 10.1164/rccm.200310-1436ST 15355871

[15] Raghu G Remy-Jardin M Ryerson CJ Myers JL Kreuter M Vasakova M. Diagnosis of hypersensitivity pneumonitis in adults. An official ATS/JRS/ALAT clinical practice guideline. Am J Respir Crit Care Med. (2020) 202:e36–69. doi: 10.1164/rccm.202005-2032ST, PMID: 32706311 PMC7397797

[16] Ryerson CJ Hartman T Elicker BM Ley B Lee JS Abbritti M. Clinical features and outcomes in combined pulmonary fibrosis and emphysema in idiopathic pulmonary fibrosis. Chest. (2013) 144:234–40. doi: 10.1378/chest.12-2403, PMID: 23370641

[17] Ponikowski P Voors AA Anker SD Bueno H Cleland JGF Coats AJS. 2016 ESC guidelines for the diagnosis and treatment of acute and chronic heart failure: the task force for the diagnosis and treatment of acute and chronic heart failure of the European Society of Cardiology (ESC) developed with the special contribution of the Heart Failure Association (HFA) of the ESC. Eur Heart J. (2016) 37:2129–200. doi: 10.1093/eurheartj/ehw128, PMID: 27206819

[18] Miller MR Hankinson J Brusasco V Burgos F Casaburi R Coates A. Standardisation of spirometry. Eur Respir J. (2005) 26:319–38. doi: 10.1183/09031936.05.00034805 16055882

[19] Macintyre N Crapo RO Viegi G Johnson DC van der Grinten CPM Brusasco V. Standardisation of the single-breath determination of carbon monoxide uptake in the lung. Eur Respir J. (2005) 26:720–35. doi: 10.1183/09031936.05.00034905, PMID: 16204605

[20] Ryerson CJ Vittinghoff E Ley B Lee JS Mooney JJ Jones KD. Predicting survival across chronic interstitial lung disease: the ILD-GAP model. Chest. (2014) 145:723–8. doi: 10.1378/chest.13-1474, PMID: 24114524

[21] Wells AU Desai SR Rubens MB Goh NSL Cramer D Nicholson AG. Idiopathic pulmonary fibrosis: a composite physiologic index derived from disease extent observed by computed tomography. Am J Respir Crit Care Med. (2003) 167:962–9. doi: 10.1164/rccm.2111053 12663338

[22] Meyer KC Raghu G Baughman RP Brown KK Costabel U du Bois RM. An official American Thoracic Society clinical practice guideline: the clinical utility of bronchoalveolar lavage cellular analysis in interstitial lung disease. Am J Respir Crit Care Med. (2012) 185:1004–14. doi: 10.1164/rccm.201202-0320ST, PMID: 22550210

[23] Lange P Celli B Agustí A Boje Jensen G Divo M Faner R. Lung-function trajectories leading to chronic obstructive pulmonary disease. N Engl J Med. (2015) 373:111–22. doi: 10.1056/NEJMoa1411532, PMID: 26154786

[24] Keskitalo E Salonen J Vähänikkilä H Kaarteenaho R. Survival of patients with asbestosis can be assessed by risk-predicting models. Occup Environ Med. (2021) 78:516–21. doi: 10.1136/oemed-2020-106819, PMID: 33637623

[25] Ma R Li S Wang Y Yang S Bao N Ye Q. High-resolution computed tomography features of asbestosis versus fibrotic hypersensitivity pneumonitis: an observational study. BMC Pulm Med. (2022) 22:207. doi: 10.1186/s12890-022-01967-3, PMID: 35614422 PMC9131664

[26] Enomoto Y Takemura T Hagiwara E Iwasawa T Okudela K Yanagawa N. Features of usual interstitial pneumonia in patients with primary Sjögren’s syndrome compared with idiopathic pulmonary fibrosis. Respir Investig. (2014) 52:227–35. doi: 10.1016/j.resinv.2014.02.003, PMID: 24998369

[27] Roggli VL Gibbs AR Attanoos R Churg A Popper H Cagle P. Pathology of asbestosis-an update of the diagnostic criteria: report of the asbestosis committee of the college of American Pathologists and Pulmonary Pathology Society. Arch Pathol Lab Med. (2010) 134:462–80. doi: 10.5858/134.3.462, PMID: 20196674

[28] Mäkelä K Mäyränpää MI Sihvo H-K Bergman P Sutinen E Ollila H. Artificial intelligence identifies inflammation and confirms fibroblast foci as prognostic tissue biomarkers in idiopathic pulmonary fibrosis. Hum Pathol. (2021) 107:58–68. doi: 10.1016/j.humpath.2020.10.008, PMID: 33161029

[29] Wang P Jones KD Urisman A Elicker BM Urbania T Johannson KA. Pathologic findings and prognosis in a large prospective cohort of chronic hypersensitivity pneumonitis. Chest. (2017) 152:502–9. doi: 10.1016/j.chest.2017.02.011, PMID: 28223152

[30] Maillet T Goletto T Beltramo G Dupuy H Jouneau S Borie R. Usual interstitial pneumonia in ANCA-associated vasculitis: a poor prognostic factor. J Autoimmun. (2020) 106:102338. doi: 10.1016/j.jaut.2019.102338, PMID: 31570253

[31] Kim EJ Elicker BM Maldonado F Webb WR Ryu JH Van Uden JH. Usual interstitial pneumonia in rheumatoid arthritis-associated interstitial lung disease. Eur Respir J. (2010) 35:1322–8. doi: 10.1183/09031936.00092309 19996193

[32] Watanabe T Minezawa T Hasegawa M Goto Y Okamura T Sakakibara Y. Prognosis of pulmonary fibrosis presenting with a usual interstitial pneumonia pattern on computed tomography in patients with myeloperoxidase anti-neutrophil cytoplasmic antibody-related nephritis: a retrospective single-center study. BMC Pulm Med. (2019) 19:194. doi: 10.1186/s12890-019-0969-5, PMID: 31675941 PMC6824021

[33] Richeldi L Costabel U Selman M Kim DS Hansell DM Nicholson AG. Efficacy of a tyrosine kinase inhibitor in idiopathic pulmonary fibrosis. N Engl J Med. (2011) 365:1079–87. doi: 10.1056/NEJMoa1103690 21992121

[34] Richeldi L Cottin V du Bois RM Selman M Kimura T Bailes Z. Nintedanib in patients with idiopathic pulmonary fibrosis: combined evidence from the TOMORROW and INPULSIS^®^ trials. Respir Med. (2016) 113:74–9. doi: 10.1016/j.rmed.2016.02.001, PMID: 26915984

[35] Raghu G Weycker D Edelsberg J Bradford WZ Oster G. Incidence and prevalence of idiopathic pulmonary fibrosis. Am J Respir Crit Care Med. (2006) 174:810–6. doi: 10.1164/rccm.200602-163OC 16809633

[36] Lu Y Chen J Wang S Tian Z Fan Y Wang M. Identification of genetic signature associated with aging in pulmonary fibrosis. Front Med. (2021) 8:744239. doi: 10.3389/fmed.2021.744239, PMID: 34746180 PMC8564051

[37] Kim H Cho S-K Song Y-J Kang J Jeong S-A Kim HW. Clinical characteristics of rheumatoid arthritis patients with interstitial lung disease: baseline data of a single-center prospective cohort. Arthritis Res Ther. (2023) 25:43. doi: 10.1186/s13075-023-03024-8, PMID: 36932433 PMC10022152

[38] Toyoda Y Koyama K Kawano H Nishimura H Kagawa K Morizumi S. Clinical features of interstitial pneumonia associated with systemic lupus erythematosus. Respir Investig. (2019) 57:435–43. doi: 10.1016/j.resinv.2019.04.005 31235450

[39] Wang L-J Cai H-R Xiao Y-L Wang Y Cao M-S. Clinical characteristics and outcomes of hypersensitivity pneumonitis: a population-based study in China. Chin Med J. (2019) 132:1283–92. doi: 10.1097/CM9.0000000000000256, PMID: 31021982 PMC6629344

[40] Laurent F Benlala I Dournes G Gramond C Thaon I Clin B. Interstitial lung abnormalities detected by CT in asbestos-exposed subjects are more likely associated to age. J Clin Med. (2021) 10:3130. doi: 10.3390/jcm10143130, PMID: 34300298 PMC8307087

[41] Yunt ZX Chung JH Hobbs S Fernandez-Perez ER Olson AL Huie TJ. High resolution computed tomography pattern of usual interstitial pneumonia in rheumatoid arthritis-associated interstitial lung disease: relationship to survival. Respir Med. (2017) 126:100–4. doi: 10.1016/j.rmed.2017.03.027, PMID: 28427540

[42] Moll R Franke WW Schiller DL Geiger B Krepler R. The catalog of human cytokeratins: patterns of expression in normal epithelia, tumors and cultured cells. Cells. (1982) 31:11–24. doi: 10.1016/0092-8674(82)90400-7, PMID: 6186379

[43] Molyneaux PL Fahy WA Byrne AJ Braybrooke R Saunders P Toshner R. CYFRA 21-1 predicts progression in idiopathic pulmonary fibrosis: a prospective longitudinal analysis of the PROFILE cohort. Am J Respir Crit Care Med. (2022) 205:1440–8. doi: 10.1164/rccm.202107-1769OC, PMID: 35363592 PMC9875897

[44] Warheit-Niemi HI Huizinga GP Edwards SJ Wang Y Murray SK O’Dwyer DN. Fibrotic lung disease alters neutrophil trafficking and promotes neutrophil elastase and extracellular trap release. Immunohorizons. (2022) 6:817–34. doi: 10.4049/immunohorizons.2200083, PMID: 36534439 PMC10542701

[45] Gregory AD Kliment CR Metz HE Kim K-H Kargl J Agostini BA. Neutrophil elastase promotes myofibroblast differentiation in lung fibrosis. J Leukoc Biol. (2015) 98:143–52. doi: 10.1189/jlb.3HI1014-493R, PMID: 25743626 PMC4763951

[46] Veeraraghavan S Latsi PI Wells AU Pantelidis P Nicholson AG Colby TV. BAL findings in idiopathic nonspecific interstitial pneumonia and usual interstitial pneumonia. Eur Respir J. (2003) 22:239–44. doi: 10.1183/09031936.03.00105202 12952254

[47] Wells AU Brown KK Flaherty KR Kolb M Thannickal VJ. What’s in a name? That which we call IPF, by any other name would act the same. Eur Respir J. (2018) 51:1800692. doi: 10.1183/13993003.00692-2018, PMID: 29773608

[48] Karampitsakos T Juan-Guardela BM Tzouvelekis A Herazo-Maya JD. Precision medicine advances in idiopathic pulmonary fibrosis. EBioMedicine. (2023) 95:104766. doi: 10.1016/j.ebiom.2023.104766, PMID: 37625268 PMC10469771

[49] Greiner S Jud A Aurich M Hess A Hilbel T Hardt S. Reliability of noninvasive assessment of systolic pulmonary artery pressure by Doppler echocardiography compared to right heart catheterization: analysis in a large patient population. J Am Heart Assoc. (2014) 3:e001103. doi: 10.1161/JAHA.114.001103, PMID: 25146706 PMC4310406

