# Supplementary material for: Disease progression in patients with usual interstitial pneumonia and probable UIP patterns on computed tomography with various underlying etiologies: a retrospective cohort study
Source: Front Med (Lausanne). 2023 Oct 12;10:1246767. doi: 10.3389/fmed.2023.1246767 (PMC10601466; doi:10.3389/fmed.2023.1246767)
Supplement: Supplementary file 1 [file Table_1.DOCX]

**Table S1.** Comparisons of different types of CTD-UIP

|  | RA-UIP | pSS-UIP | AAV-UIP | other CTD-UIP | *P* value |
| --- | --- | --- | --- | --- | --- |
| N | 52 | 71 | 58 | 48 |  |
| Age | 63.9 ± 9.1 | 66.4 ± 8.9 | 69.4 ± 8.8^ac^ | 62.2 ± 10.3 | 0.001 |
| Male, n (%) | 35 (67.3) | 35 (49.3) | 36 (62.1) | 27 (56.3) | 0.212 |
| BMI, kg/m^2^ | 23.6 ± 3.6 | 23.7 ± 3.8 | 23.0 ± 3.6 | 23.5 ± 3.7 | 0.747 |
| Ever smoker, n (%) | 27 (51.9) | 36 (50.7) | 33 (56.9) | 21 (43.7) | 0.616 |
| UIP/probable UIP | 43/9 | 54/17 | 46/12 | 35/13 | 0.661 |
| Pulmonary function |  |  |  |  |  |
| FVC, L | 2.9 ± 0.8 | 2.5 ± 0.7 | 2.9 ± 0.9 | 2.7 ± 0.8 | 0.074 |
| FEV1% predicted | 86.8±16.5 | 89.6±23.6 | 89.9±16.5 | 87.3±21.9 | 0.873 |
| FVC% predicted | 85.9 ± 18.1 | 88.6 ± 23.2 | 91.3 ± 20.3 | 87.5 ± 22.7 | 0.781 |
| DLCOsb% predicted | 56.2 ± 19.9 | 49.9 ± 18.1 | 53.5 ± 19.7 | 52.3 ± 21.1 | 0.503 |
| GAP score | 2.0 (1.0-4.0) | 3.0 (2.0-4.0) | 3.0 (2.0-4.0) | 2.5 (1.0-4.0) | 0.465 |
| CPI score | 37.9 ± 16.2 | 42.1 ± 14.6 | 38.4 ± 17.6 | 40.3 ± 17.9 | 0.628 |
| Blood cytology |  |  |  |  |  |
| Leucocytes, ×10^9^/L | 7.2 ± 1.8 | 5.9 ± 1.7 | 8.2 ± 2.6^bc^ | 6.5 ± 2.2 | < 0.001 |
| Neutrophils, ×10^9^/L | 4.8 ± 1.5 | 3.3 ± 1.3 | 6.0 ± 2.4^abc^ | 3.9 ± 1.6 | < 0.001 |
| Lymphocytes, ×10^9^/L | 1.9 ± 0.8 | 1.9 ± 0.6 | 1.5 ± 0.6^abc^ | 1.9 ± 0.7 | 0.006 |
| Monocytes, ×10^9^/L | 0.5 ± 0.2 | 0.4 ± 0.2 | 0.5 ± 0.2 | 0.4 ± 0.2 | 0.114 |
| ESR, mm/h | 28.0 (14.0-50.0) | 20.0 (10.8-39.3) | 46.0 (27.8-58.8)^abc^ | 19.0 (13.0-50.0) | < 0.001 |
| CRP, mg/dL | 0.8 (0.4-0.7) | 0.5 (0.2-0.9) | 3.6 (0.9-8.9)^abc^ | 0.5 (0.3-0.8) | < 0.001 |
| IgG, mg/dL | 1638.4 ± 390.5 | 1687.1 ± 615.7 | 1566.0 ± 388.5 | 1750.9 ± 597.5 | 0.324 |
| Serum oncomarkers | 49 (94.2) | 64 (90.1) | 56 (96.6) | 43 (89.6) | 0.394 |
| CYFRA21-1, ng/ml | 2.8 (1.9-3.6) | 2.7 (2.5-4.0) | 3.0 (2.0-4.5) | 3.5 (2.7-4.4) | 0.229 |
| CEA, ng/ml | 2.2 (1.3-3.2) | 2.5 (1.8-3.8) | 2.4 (1.2-3.8) | 2.6 (1.7-4.1) | 0.436 |
| CA19-9, U/ml | 31.1 (15.4-82.9) | 19.2 (8.9-68.2) | 16.1 (8.3-29.2) | 26.2 (11.9-85.9) | 0.081 |
| CA125, U/ml | 26.2 (17.1-47.2) | 24.5 (12.2-43.9) | 30.8 (14.8-55.6) | 16.0 (9.2-37.4) | 0.139 |
| BALF cytology | 24 (46.2) | 31 (43.7) | 28 (48.3) | 25 (52.1) | 0.831 |
| Macrophages, % | 69.0 (54.3-74.8) | 52.5 (33.5-70.8) | 55.0 (19.0-80.0) | 59.5 (47.0-66.5) | 0.284 |
| Lymphocytes, % | 5.5 (3.3-7.0) | 4.5 (1.3-9.0) | 4.0 (0.0-5.0) | 5.5 (2.3-8.5) | 0.703 |
| Neutrophils, % | 23.5 (17.3-38.3) | 35.0 (20.0-59.5) | 35.0 (17.0-75.0) | 31.0 (20.3-48.9) | 0.433 |
| Eosinophils, % | 1.0 (0.3-2.8) | 1.0 (0-2.0) | 0.5 (0-1.0) | 1.0 (1.0-4.5) | 0.284 |
| Emphysema, n (%) | 14 (26.9) | 21 (29.6) | 18 (31.0) | 10 (20.8) | 0.657 |
| Pulmonary hypertension, n (%) | 9 (17.3) | 17 (23.9) | 5 (8.6) | 12 (25.0) | 0.090 |
| Follow-up time (month) | 46.1 (18.7-64.6) | 52.9 (25.2-91.5) | 45.7 (14.7-67.1) | 46.7 (21.4-66.1) | 0.161 |
| Treatment, n (%) |  |  |  |  |  |
| Immunosuppressive therapy | 44 (84.6) | 52 (73.2) | 53 (91.4) | 38 (79.2) | 0.055 |
| Antifibrotic therapy | 8 (15.4) | 9 (12.7) | 4 (6.9) | 7 (14.6) | 0.531 |
| Lung transplantation | 2 (3.8) | 0 (0) | 1 (1.7) | 0 (0) | 0.239 |
| ΔFVC, mL/year | 88.1 (6.7-265.4)^bc^ | 25.9 (-138.1-162.6) | 72.9 (-35.6-257.4)^bc^ | 20.7 (-158.9-132.2) | 0.002 |
| Cumulative TFS, % |  |  |  |  |  |
| One year | 90.4% | 92.9% | 77.6% | 97.9% | — |
| Three years | 70.3% | 75.2% | 61.5% | 80.0% | — |
| Five years | 46.9% | 69.0% | 40.0% | 64.9% | — |

Data were presented as mean ± SD or median (IQR) or n (%).

^a^*P* < 0.0083 versus the RA group, ^b^*P* < 0.0083 versus the pSS group, ^c^*P* < 0.0083 versus the other CTD-UIP group.

CTD, connective tissue disease; UIP, usual interstitial pneumonia; RA, rheumatoid arthritis; pSS, primary Sjögren's syndrome; AAV, anti-neutrophil cytoplasmic antibody associated vasculitis; BMI, body mass index; FVC, forced vital capacity; FEV1, forced expiratory volume in the first second; DLCOsb, single-breath diffusing capacity of the lung for carbon monoxide; GAP, gender-age-physiology; CPI, composite physiologic index; ESR, cells erythrocyte sedimentation rate; CRP, C-reactive protein; IgG, immunoglobulin G; CYFRA21-1, cytokeratin fraction 21-1; CEA, carcinoembryonic antigen; CA, carbohydrate antigen; BALF, bronchoalveolar lavage fluid; TFS, transplant-free survival.

**Table S2.** FVC decline and TFS compared between patients with and without emphysema

|  | N | Baseline FVC, L | Annual FVC decline, mL | TFS, month | *P*1 value | *P*2 value |
| --- | --- | --- | --- | --- | --- | --- |
| IPF | 320 | 2.9 ± 0.8 | 133.9 (39.3-269.1) | 55.9 |  |  |
| Emphysema | 112 | 3.1 ± 0.8 | 50.7 (19.1-176.5) | 65.6 | 0.047 | 0.384 |
| Without emphysema | 208 | 2.8 ± 0.7 | 177.3 (44.5-279.3) | 54.2 |  |  |
| CTD-UIP | 229 | 2.7 ± 0.8 | 24.5 (-72.8-158.9) | 66.7 |  |  |
| Emphysema | 63 | 3.1 ± 0.7 | -35.6 (-250.9-50.8) | 70.9 | 0.024 | 0.868 |
| Without emphysema | 166 | 2.6 ± 0.8 | 38.9 (-16.8-191.5) | 65.2 |  |  |

Data were presented as mean ± SD or median (IQR) or median.

*P*1 value: *P* value derived from annual FVC decline comparison between patients with and without emphysema; *P*2 value: *P* value derived from TFS comparison between patients with and without emphysema.

FVC, forced vital capacity; TFS, transplant-free survival; IPF, idiopathic pulmonary fibrosis; CTD, connective tissue disease; UIP, usual interstitial pneumonia.

**Table S3.** FVC decline and TFS compared between patients with and without PHT

|  | N | Baseline FVC, L | Annual FVC decline, mL | TFS, month | *P1* value | *P*2 value |
| --- | --- | --- | --- | --- | --- | --- |
| IPF | 320 | 2.9 ± 0.8 | 133.9 (39.3-269.1) | 55.9 |  |  |
| PHT | 56 | 2.7 ± 0.9 | 162.6 (43.6-259.3) | 35.9 | 0.708 | < 0.001 |
| Without PHT | 264 | 3.0 ± 0.8 | 105.0 (25.2-273.6) | 65.2 |  |  |
| CTD-UIP | 229 | 2.7 ± 0.8 | 24.5 (-72.8-158.9) | 66.7 | 0.827 | < 0.001 |
| PHT | 43 | 2.3 ± 0.7 | 12.7 (-225.0-272.7) | 28.5 |  |  |
| Without PHT | 186 | 2.8 ± 0.8 | 33.0 (-48.5-150.4) | 80.9 |  |  |

Data were presented as mean ± SD or median (IQR) or median.

*P*1 value: *P* value derived from annual FVC decline comparison between patients with and without PHT; *P*2 value: *P* value derived from TFS comparison between patients with and without PHT.

FVC, forced vital capacity; TFS, transplant-free survival; PHT, pulmonary hypertension; IPF, idiopathic pulmonary fibrosis; CTD, connective tissue disease; UIP, usual interstitial pneumonia.
